# Qualitative study to characterize patient experience and relevance of patient-reported outcome measures for patients with metastatic synovial sarcoma

**DOI:** 10.1186/s41687-022-00450-1

**Published:** 2022-05-04

**Authors:** Laurie Eliason, Laura Grant, Anya Francis, Anna Cardellino, Ken Culver, Sant P. Chawla, Rob Arbuckle, Shibani Pokras

**Affiliations:** 1 Value Evidence & Outcomes, GlaxoSmithKline, Collegeville, PA 19426 USA; 2 Patient-Centered Outcomes, Adelphi Values, Bollington, Cheshire, SK10 5JB UK; 3 Sarcoma Oncology Center, Santa Monica, CA 90403 USA

**Keywords:** Qualitative interviews, Synovial sarcoma, Metastatic, PRO, HRQoL, Symptoms, Impact, Content validity

## Abstract

**Background:**

The outlook for patients with metastatic synovial sarcoma (mSS) is poor. Better understanding of patient experience in this setting, beyond clinical measures, may guide improvements in management. Validated patient-reported outcome (PRO) instruments specific to many types of cancer exist, but for rare cancers this is often not the case.

**Methods:**

This study aimed to characterize patient experiences of symptoms and impacts of mSS and evaluate the content validity and relevance of the novel European Organization for Research and Treatment of Cancer Item Library 31 (EORTC IL31) Disease Symptoms PRO tool assessing synovial sarcoma symptoms. This tool comprises items from preexisting, validated cancer-specific PRO instruments from the EORTC Item Library. It was developed as an mSS-specific add-on to the EORTC Quality of Life Questionnaire Core 30 (QLQ-C30), which evaluates general cancer and treatment-related symptoms and functioning. This was a non-interventional, qualitative interview study involving semi-structured, concept elicitation (CE) and cognitive debriefing (CD) telephone interviews in adults with mSS. CE explored symptoms and their impact on functioning and quality of life; CD assessed participant understanding and relevance of the PRO tools.

**Results:**

Among the 8 participants, the most common disease-related symptoms reported during CE were fatigue and pain, while shortness of breath was one of the most bothersome. The greatest negative impacts of mSS occurred in domains of physical functioning and sleep. Key treatment priorities for patients were to improve disrupted sleep and ability to undertake strenuous activities.

**Conclusions:**

The interviews showed that, when used together, the EORTC IL31 and EORTC QLQ-C30 covered symptoms and impacts of most relevance and importance to patients with mSS, with no notable gaps and good conceptual coverage. This study therefore supports the content validity of 2 tools in mSS, advocating their use in clinical trials to assess treatment impact on PRO measures of importance to these patients.

**Supplementary Information:**

The online version contains supplementary material available at 10.1186/s41687-022-00450-1.

## Background

Synovial sarcoma (SS) is a rare form of soft-tissue sarcoma (STS). It accounts for approximately 5%–10% of cases of STS, which in turn comprise < 1% of all new cancer diagnoses in the USA [1, 2]. Indeed, the estimated incidence of SS in the USA is only 800–1000 cases [3]. SS is an aggressive malignancy that typically develops in the soft tissues of the arm, leg or foot, or near joints such as the wrist, knee, or ankle, although it can also affect the lung or abdomen [1, 4]. This cancer may be seen in any age group, with an average age at diagnosis of around 40 years [1]. SS is associated with local recurrences, and distant metastases occur in 50–70% of patients, with pulmonary metastases being most likely [5]. The outlook for patients with metastatic SS (mSS) is poor, with limited treatment options, reduced health-related quality of life (HRQoL), and overall survival of only 8.8–21.7 months [1, 6, 7].

A better understanding of the patient experience of mSS—beyond the information gleaned from clinical measures such as treatment response, progression rate, and survival—may help evaluate risks and benefits of potential new treatments and guide improvements in management. This includes characterizing the impact of the disease and its treatment on a patient’s symptoms, functioning, and HRQoL in clinical trials and other research studies. In metastatic cancer settings, where treatments may not substantially extend life or achieve a meaningful remission, consideration of the impact of treatment on the ‘quality’ of life versus the ‘quantity’ of life is particularly important. However, to date, little qualitative patient research has been undertaken in mSS, due to the rare and debilitating nature of this condition [1]. Typically, available information on disease burden and HRQoL in these patients is restricted to studies of STS: a broader classification that includes multiple histologies in addition to that of SS [6, 8–12]. Research in this area has also been hampered by the lack of an SS-specific patient-reported outcome (PRO) instrument that can collect valuable information on symptoms and relevant domains of functioning for patients with metastatic disease. In the aforementioned studies in the broader category of STS, functioning and HRQoL have typically been explored using generic instruments. These include HRQoL questionnaires, such as Short-Form-36 (SF-36), EuroQoL 5-dimension scale (EQ-5D), or European Organization for Research and Treatment of Cancer Quality of Life Questionnaire Core 30 (EORTC QLQ-C30); or generic measures of cancer symptoms such as the MD Anderson Symptom Inventory (MDASI), Memorial Symptom Assessment Scale (MSAS), or 3-item Cancer-Related Symptoms Questionnaire [8, 10, 13]. These tools may cover broad domains of interest, but they are not specific to considerations of relevance to SS [14]. They do not inform on which symptoms and impacts are most burdensome for patients with mSS, and which would be most important to improve through intervention [14].

When examining PROs, it is important to utilize instruments that are fit-for-purpose and evaluate concepts that are relevant to patients with the condition of interest. Although there are well-developed and validated available PROs for many types of cancers, additional research may be needed to identify measures that are suitable for rare cancers, as is the case for mSS. The EORTC Item Library, an online platform containing all EORTC HRQoL items, has been developed to allow for flexibility in the inclusion of additional concepts of importance in the setting of novel therapies or rare cancers that are not included in core sets of PRO questionnaires [15].

The novel EORTC Item Library 31 (EORTC IL31) Disease Symptoms PRO instrument comprises 10 symptom items relating to SS selected from preexisting, validated EORTC PRO instruments as part of the EORTC Item Library, namely the EORTC Quality of Life Questionnaire – Chemotherapy-Induced Peripheral Neuropathy 20-item scale (EORTC QLQ-CIPN20) and the EORTC Quality of Life Questionnaire – Lung Cancer module (EORTC QLQ-LC13). The EORTC IL31 was developed to be used as an mSS-specific add-on to the EORTC QLQ-C30, which evaluates general cancer and treatment-related symptoms and functioning. The selected items included in the EORTC IL31 PRO instrument are intended to cover symptoms considered most likely to be relevant to the patient population with mSS and were selected based on clinical input and literature review (Additional file 1: Content).

For any PRO measure intended for inclusion in clinical trials, the US Food and Drug Administration (FDA) strongly recommends establishing content validity using individual interviews or focus groups involving qualitative concept elicitation (CE) research followed by cognitive debriefing (CD) with relevant stakeholders such as patients with the disease of interest [16]. The CE should start with open-ended questioning to explore which aspects of the disease/condition are most important to patients and how patients describe those concepts. Such CE research is typically performed prior to drafting a new PRO measure but is also important when existing PRO items are used in a new ‘Context of Use’ (ie, a new condition or population), to confirm relevance of the concepts being assessed. In the second step, CD, the content validity of any such tool must be rigorously evaluated to ensure that patients with the disease of interest understand the PRO instrument as intended. This step includes assessment of whether the instructions, items, and response scale are worded in a manner that is clear and interpreted consistently, and provides further confirmation of the relevance and comprehensiveness of the concepts within the proposed tool [16].

The objectives of this study were to characterize patient experiences of symptoms and impacts of mSS through CE questioning and CD interviewing, and to evaluate the content validity and relevance of EORTC IL31 when used alongside the existing core cancer PRO measure, EORTC QLQ-C30, to measure these symptoms and impacts in a clinical trial setting.

## Methods

### Study design and participants

This was a non-interventional, qualitative interview study involving adults with a clinician-confirmed diagnosis of mSS. Interviews were conducted in the USA between October 2019 and February 2020 by individuals trained in qualitative interviewing methods. Participants were fluent English-speaking men and women aged ≥ 18 years at the time of the interview with no significant physical, cognitive, psychological, or psychiatric condition that would limit their ability to participate (other than mSS); and no other prior malignancy not in remission. Given the rarity of the patient population, all participants were identified by and recruited from the Sarcoma Oncology Center, California, USA, which specializes in sarcoma treatment. Quota sampling for age (n = 4 < 25 years old, n = 4 ≥ 25 years old), sex (n = 5 male, n = 5 female), and location of metastasis (n = 2 non-pulmonary) was applied to ensure participants with a range of demographic and clinical characteristics were recruited. Between 10 and 12 participants were targeted for inclusion with the aim of interviewing enough participants to support requirements for assessing conceptual saturation but keeping the target sample size low, with consideration of how rare mSS is and how unwell the target population typically is. All participants provided written informed consent before enrolment. The study was approved and overseen by the Western Independent Review Board.

**Fig. 1:**
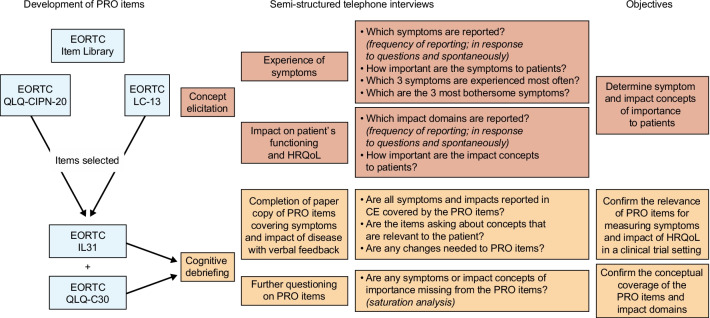
Summary of Study Methods and Objectives. CE, concept elicitation; EORTC, European Organization for Research and Treatment of Cancer; HRQoL, health-related quality of life; IL31, Item Library 31; PRO, patient-reported outcomes; QLQ-C30, Quality of Life Questionnaire Core 30; QLQ-CIPN20, Quality of Life Questionnaire—Chemotherapy-Induced Peripheral Neuropathy 20-item scale

The study consisted of semi-structured, combined CE and CD telephone interviews lasting 60–90 min (Fig. 1). The CE questioning began with broad open-ended questions to explore experiences of symptoms of mSS and how these affected each participant’s life, followed by more focused questions to probe areas of interest that were not mentioned spontaneously or to further clarify concepts/statements. Participants were asked to discuss symptoms experienced at the time of the interview and more generally, including before and following treatment. These symptoms could include treatment side effects.

For the CD questioning, participants read and completed paper copies of the EORTC IL31 and QLQ-C30 PRO items. The EORTC IL31 comprised 10 EORTC symptom items originating from the EORTC-QLQ-CIPN20 and the EORTC-QLQ-LC13 of the EORTC Item Library. The 30 items of the EORTC QLQ-C30 comprised 9 symptom items, 19 impact items, and 2 global HRQoL items. These global HRQoL items asked participants to rate their overall health (item 29) and overall HRQoL (item 30) in the past week on a 7-point scale (0 = ‘very poor’ to 7 = ‘excellent’). Tables 1 and 2 summarize the symptom and impact items reviewed by participants.

**Table 1:** Symptom items used in the interviews

EORTC source	Concept	Question
EORTC IL31 Items (from EORTC-QLQ-CIPN20)	Nerve/shooting/burning pain	1. Did you have shooting or burning pain in your fingers or hands?
		2. Did you have shooting or burning pain in your toes or feet?
EORTC IL31 Items (from EORTC-QLQ-LC13)	Coughing	3. How much did you cough?
	Coughing up blood	4. Did you cough up blood?
	Shortness of breath	5. Were you short of breath when rested?
		6. Were you short of breath when you walked?
		7. Were you short of breath when you climbed stairs?
	Pain (chest, arms, shoulder)	8. Have you had pain in your chest?
		9. Have you had pain in your arm or shoulder?
	Pain	10. Have you had pain in other parts of your body?
EORTC QLQ-C30	Shortness of breath	8. Were you short of breath?
	Pain	9. Have you had pain?
	Fatigue	10. Did you need to rest?
		18. Were you tired?
		12. Have you felt weak?
	Gastrointestinal symptoms	14. Have you felt nauseated?
		16. Have you been constipated?
		15. Have you vomited?
		17. Have you had diarrhea?

C30, Core 30; CIPN20, Chemotherapy-Induced Peripheral Neuropathy 20-item scale; EORTC, European Organization for Research and Treatment of Cancer; IL31, Item Library 31; LC13, Lung Cancer 13; QLQ, Quality of Life Questionnaire

**Table 2:** Impact items used in the interviews

EORTC source	Concept	Question
EORTC QLQ-C30	Physical functioning	1. Do you have any trouble doing strenuous activities, like carrying a heavy shopping bag or suitcase?
		2. Do you have any trouble taking a long walk?
		3. Do you have any trouble taking a short walk outside of the house?
		4. Do you need to stay in bed or a chair during the day?
	Sleep	11. Have you had trouble sleeping?
	ADL and work	6. Were you limited in doing either your work or other daily activities?
		19. Did pain interfere with your daily activities?
	Emotional	21. Did you feel tense?
		22. Did you worry?
		23. Did you feel irritable?
		24. Did you feel depressed?
	Social	26. Has your physical condition or medical treatment interfered with your family life?
		27. Has your physical condition or medical treatment interfered with your social activities?
	Appetite	13. Have you lacked appetite?
	Hobbies	7. Were you limited in pursuing your hobbies or other leisure activities?
	Financial	28. Has your physical condition or medical treatment caused you financial difficulties?
	Cognition	20. Have you had difficulty concentrating on things, like reading a newspaper or watching television?
		25. Have you had difficulty remembering things?
	Personal care	5. Do you need help with eating, dressing, washing yourself or using the toilet?

ADL, activities of daily living; EORTC QLQ-C30, European Organization for Research and Treatment of Cancer Quality of Life Questionnaire Core 30

After completing the PRO questionnaires, participants were asked detailed questions about the relevance of the PRO items, the response options, and the recall periods. Participants were also asked to rate how important it would be to see an improvement in each of the symptom and impact concepts on a 4-point verbal descriptor scale (0 = ‘not at all important’, 1 = ‘a little important’, 2 = ‘important’ and 3 = ‘very important’). Given the limited time available, the CD questioning focused on evaluating relevance to the patient sample, with limited questioning specifically focused on comprehension. This focus reflects the fact that the items being tested were taken from existing measures for which understanding and consistent interpretation by patients has been well-established. Moreover, to avoid undue patient burden, extending the interviews was not considered appropriate given how unwell the patients were.

### Data analysis

All interviews were audio recorded, transcribed verbatim, and coded using ATLAS.ti software and thematic analysis and framework approach methods [17]. A conceptual model was developed to summarize symptoms and impacts associated with mSS, based on the literature review and key findings from the CE part of the interviews.

Conceptual saturation for all domains relevant to higher-level concepts of importance was confirmed through saturation analysis. Participant transcripts were ordered chronologically and divided into 3 groups; findings from each group of interviews were compared iteratively. Concepts that emerged in the first set of interviews were compared with the concepts reported in the second set of interviews. The consolidated list of concepts elicited from the first 2 sets of participants were then compared to the concepts elicited in the third set. At each step, any new concepts that had not emerged in the previous set of interviews were highlighted. Saturation was deemed to be achieved when no new concepts emerged in the third, final group of interviews. Concepts identified during CE were then mapped onto the PRO items to assess conceptual coverage of key symptoms of mSS.

## Results

### Study participants

Of 14 participants consented and recruited, 6 were not included in the interviews (2 did not respond to interview scheduling requests despite repeated attempts, 2 died, 1 was too unwell to participate, 1 withdrew consent). Therefore, the interviewed sample comprised 8 participants (5 men and 3 women). The mean age of the participants was 44 years (range 21–61 years), and the sample was diverse in terms of race, ethnicity, and educational level (Table 3). Five (62.5%) participants had high-grade cancer; most participants had lung as a site of initial metastasis (87.5%), but several had ≥ 1 metastatic site. All participants had an Eastern Cooperative Oncology Group (ECOG) status of 1 and had received prior treatment, including surgery, chemotherapy, and other treatments. Seven (87.5%) participants were receiving second- or later-line treatment.

**Table 3:** Demographic and disease characteristics at time of interview

Characteristic	Participants (n = 8)
*Mean age (years), n (range)*	44 (21–61)
*Sex, n (%)*	
Men	5 (62.5)
Women	3 (37.5)
*Ethnicity, n (%)*	
Hispanic/Latino	5 (62.5)
Non-Hispanic/Latino	3 (37.5)
*Race, n (%)*	
White	4 (50)
Multi-racial	1 (12.5)
Asian, Native Hawaiian, or Pacific Islander	1 (12.5)
Mexican American	1 (12.5)
Hispanic	1 (12.5)
*Highest level of education, n (%)*	
Completed high school	3 (37.5)
College or university	1 (12.5)
Undergraduate or bachelor’s degree	2 (25.0)
Graduate degree	2 (25.0)
*Time since SS diagnosis, n (%)*	
< 2 years	2 (25.0)
2–5 years	2 (25.0)
> 5 years	4 (50.0)
*Time since mSS diagnosis, n (%)*	
< 2 years	4 (50.0)
2–5 years	2 (25.0)
> 5 years	2 (25.0)
Original site of metastasis, n (%)*	
Pulmonary	7 (87.5)
Bone	2 (25.0)
Liver	1 (12.5)
Mediastinal mass	1 (12.5)
Abdomen	1 (12.5)
Pelvis	1 (12.5)
Pancreas	1 (12.5)
Subsequent metastatic relapses, n (%)*	
Pulmonary	1 (12.5)
Right axillary mass	1 (12.5)
Anterior mediastinal	1 (12.5)
N/A	5 (62.5)
*Grade of cancer, n (%)*	
High	5 (62.5)
Low	1 (12.5)
Unknown	2 (25.0)
*Current ECOG performance status, n (%)*	
1	8 (100)
Treatment received for local SS, n (%)*	
Surgery^†^	5 (62.5)
Chemotherapy^‡^	4 (50.0)
Radiotherapy	3 (37.5)
Other (immunotherapy)	1 (12.5)
*Treatment received for mSS, n (%)*	
Chemotherapy^‡^	5 (62.5)
Surgery^†^	1 (12.5)
Other (clinical trial n = 3, immunotherapy n = 1)	4 (50.0)
Treatment received for recurrent mSS, n (%)*	
Surgery^†^	2 (25.0)
Chemotherapy^‡^	4 (50.0)
Other (clinical trial n = 5, immunotherapy n = 1, iron n = 1)	6 (75.0)
*Line of treatment for mSS, n (%)*	
2L +	7 (87.5)

2L, second line; ECOG, Eastern Cooperative Oncology Group; mSS, metastatic synovial sarcoma; SS, synovial sarcoma

^*^More than one option could be provided, thus the total is > 8

^†^Location of surgery (where specified), all n = 1 each: local SS: femur, small bowel (resection), right hand (mass resection), right elbow (below elbow amputation), right forearm, neck (mass excision), C4-C6 vertebrae (column resection); mSS: lung (lower and upper resection); recurrent mSS: lung (video-assisted thoracoscopic surgery) and lung (wedge resection)

^‡^Chemotherapy (where specified): local SS: ifosfamide (n = 2), gemcitabine, doxorubicin, and docetaxel (n = 1 each); mSS: doxorubicin, ifosfamide, and mesna (AIM; n = 2), trabectedin and nivolumab (n = 1), interferon, sargramostim, and deferolimus (n = 1 each); recurrent mSS: trabectedin and nivolumab (n = 1), trabectedin only, nivolumab only, interferon, sargramostim, zoledronic acid, atezolizumab, and anlotinib (n = 1 each)

### Symptoms and impact concepts of importance to patients

**Fig. 2:**
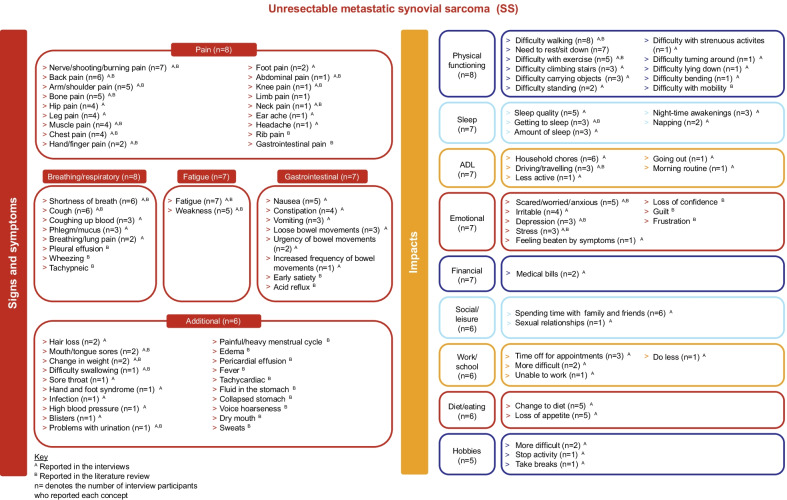
Conceptual Model of mSS. ADL, activities of daily living; mSS, metastatic synovial sarcoma

The CE interviews identified that individuals with mSS experienced numerous symptoms that could be categorized into 4 core symptom domains—pain, breathing/respiratory, fatigue, and gastrointestinal—and a fifth group of ‘additional’ symptoms including, among others, hair loss, mouth/tongue sores, and change in weight (Fig. 2). The impact of mSS was multifaceted, spanning across the 9 broad domains of physical functioning, sleep, activities of daily living (ADL), emotional, financial, social/leisure, work/school, diet/eating, and hobbies.

**Fig. 3:**
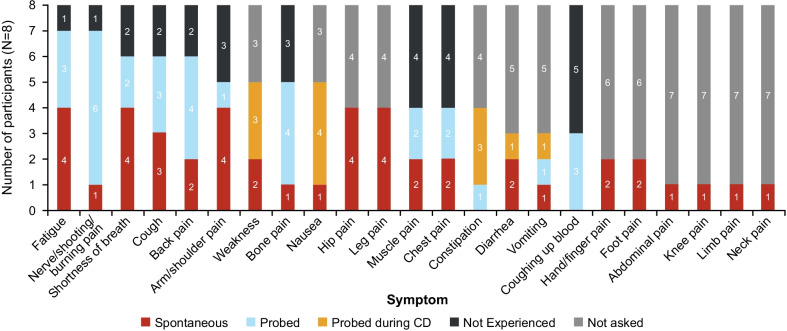
Overview of Symptoms Reported. CD, cognitive debriefing

Figure 3 provides an overview of symptoms reported during the CE phase of the interviews, detailing which symptoms were mentioned first by the participant in response to an open question (spontaneously reported) and which symptoms the participant reported experiencing only when the interviewer specifically asked about the symptom (probed). The most commonly reported symptoms (spontaneous or probed) were fatigue (*“I get pretty tired…”* [09-M-36]; *“It wears me out. It wears me out.”* [05-M-61]; Additional file 1: Table S1) and the description of nerve/shooting/burning pain in general (*“I do have a shooting pain in my fingers and hands.”* [03-M-55]; *“So that nerve pain would be kind of like the—like a spasm to me and those can be pretty painful.”* [05-M-61]), which were each reported in 7 (87.5%) participants. The specific location(s) in the body of pain experienced varied between participants. Among spontaneously reported symptoms, the most common were fatigue, shortness of breath, arm/shoulder pain, hip pain, and leg pain (each reported in 4 [50.0%] participants).

**Fig. 4:**
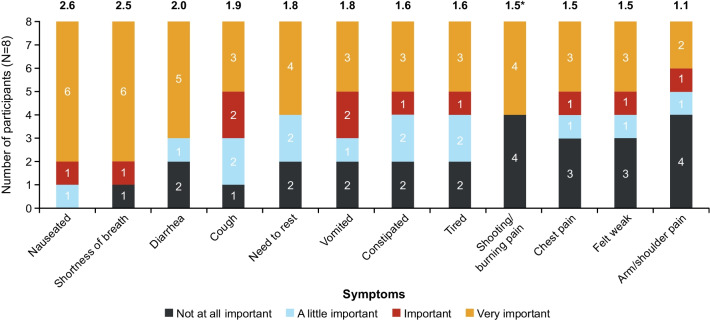
Importance Rating of Symptoms. Values at the top of each bar denote mean importance rating for that symptom generated from a scoring system of 0 = not at all important; 1 = a little important; 2 = important; 3 = very important; therefore, the higher the score, the greater the importance of the symptom

The importance ratings of symptoms mirrored the frequency of reporting. In general, the symptom concepts that were rated the most important were also the ones that were reported most frequently. The exceptions were nausea and diarrhea/loose bowel movements, which were reported by few participants (n = 4 [50.0%] and n = 3 [37.5%], respectively) but were rated with high importance. Participants most frequently reported it to be ‘very important’ for them to see an improvement in nausea and shortness of breath (n = 6 [75.0%]; mean importance rating 2.6 and 2.5, respectively, on a scale of 0–3 where 3 indicates ‘very important’) and diarrhea (n = 5 [62.5%]; mean importance rating 2.0) (Fig. 4).

In response to being asked which 3 symptoms they experienced most frequently, participants most frequently selected fatigue among the 3 reported (n = 5 [62.5%]). When asked which 3 symptoms were most bothersome, participants most frequently identified shortness of breath (n = 3 [37.5%]).

**Fig. 5:**
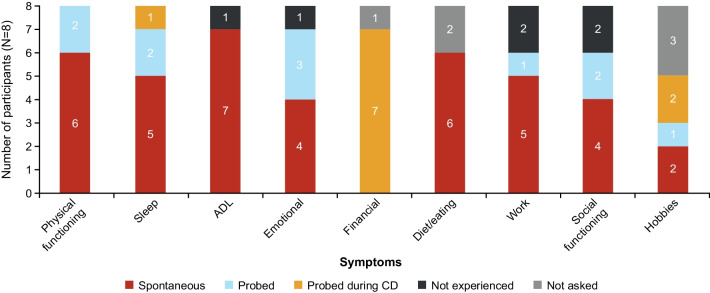
Overview of Impact Domains. ADL, activities of daily living; CD, cognitive debriefing

When questioned about how the symptoms impacted their lives, all participants reported (spontaneously [n = 6, 75.0%] or on probing [n = 2, 25.0%]) that mSS affected their physical functioning and sleep. Almost all (n = 7 [87.5%]) participants reported that it affected their ADL, emotional well-being, and finances, although finances were only mentioned when probed during CD. In addition, 75.0% of participants (n = 6) also reported an impact on domains of diet/eating, work, and social functioning (Fig. 5). Impacts on cognition (difficulty concentrating and difficulty remembering things) and personal care were perceived as less important by this cohort and were reported by relatively few participants (these concepts were not mentioned by any participant during CE). The impacts most frequently reported spontaneously were in domains of ADL (n = 7 [87.5%]), physical functioning, diet/eating (each n = 6 [75.0%]); and sleep and work (n = 5 [62.5%]).

Participants described multiple impacts on physical functioning, such as difficulty walking, exercising, climbing stairs, lifting/carrying objects, and needing to sit down/rest, with some participants avoiding activity altogether. *(“I just get a lot of shortness of breath or I, I just have to take a, take a break.”* [01-F-21]; *“…you stand up and halfway you get just a shock of bone pain.”* [14-M-36]; *“…it's hard to get comfortable when I lie down and sleep.”* [05-M-61]).

The most frequently reported sleep problem was reduced sleep quality (n = 5 [62.5%]: *“I can't get a good sleep at night…And, you know, because I'm in pain a little bit here. Then everything—it's like a combination, a combination of pain like in my leg, my arm.”* [03-M-55]; *“I can't get a full night's rest…because of the pain.”* [01-F-21]).

In terms of ADL impacts, participants most frequently reported difficulty completing household chores (n = 6 [75.0%]: *“…like sweeping and mopping, um, I do have to stop throughout it, um, to take a breath…”* [01-F-21); *“…I don’t feel that good to cook because I'm coughing…”* [12-F-43]; *“…before I was diagnosed with cancer, I was more active…I became more weak, you know, like to my activities that I did before. Now—since—ever since, since August of last year, I've been more like, uh, you know, like more like a mouse.”* [03-M-55]).

The most frequent emotional impact was participants feeling worried or anxious (n = 5 [62.5%]: *“…I worry about all my friends and family who worry about me.”* [05-M-61]; *“…you always worry if it’s ever going to come back.”* [13-F-46]); stress and depression were also common (n = 3 each; *“…I have been depressed lately…”* [01-F-21]).

**Fig. 6:**
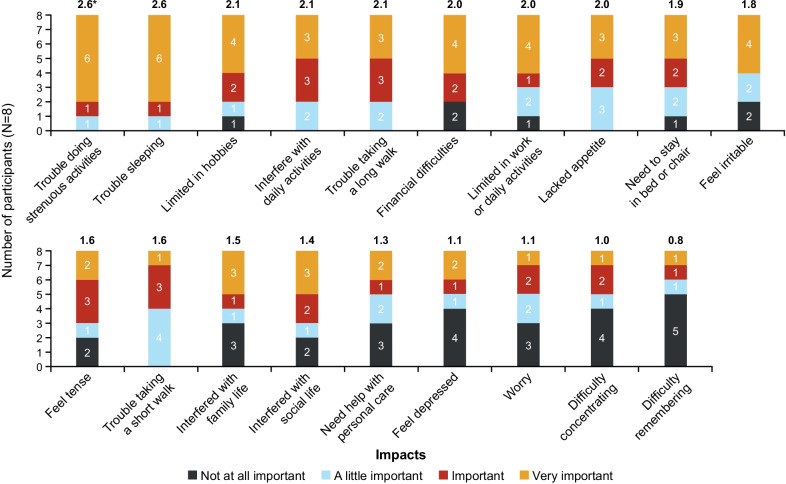
Importance Rating of Impact Concepts. *Values at the top of each bar denote mean importance rating for that symptom generated from a scoring system of 0 = not at all important; 1 = a little important; 2 = important; 3 = very important; therefore, the higher the score, the greater the importance of the impact

Importance ratings of the impact concepts revealed that improvement in ability to carry out strenuous activities and ability to sleep were the key priorities, with 6 participants (75.0%) denoting improvements in these categories to be ‘very important’. For nearly all other domains, most participants reported it would be at least ‘a little important’ to see an improvement. Aside from trouble doing strenuous activities and trouble sleeping, mean importance scores were also high (with a score of at least 2.0) for domains of trouble while taking a walk, limitations in work or daily activities, limitations in hobbies, interference with daily activities, lack of appetite, and financial difficulties (Fig. 6).

### Relevance of selected PRO items for use in a clinical trial setting

The majority of symptoms and impacts reported during the CE interviews were covered by the EORTC QLQ-C30 and EORTC IL31 PRO instruments. The exception was a small number of less-frequently reported symptoms, including problems with urination and wheezing, which are not included in either measure.

The EORTC IL31 includes multiple pain items but does not include items about pain in some of the specific locations reported in the interviews (eg, foot pain and hip pain). The EORTC-QLQ-C30 also includes a general pain domain. The EORTC IL31 includes items asking about shooting/burning pain in fingers or hands and in the toes or feet; qualitative findings indicated that while most participants experienced shooting/burning pain somewhere in their limbs (n = 7 participants [87.5%]), fewer experienced this pain specifically in their fingers, hands, toes, or feet (n = 2 participants [25.0%]), as specified in the items.

No gaps in concepts in EORTC QLQ-C30 and IL31 were identified during open-ended CE questioning. Only a small number of participants (n = 1–2 per item) suggested making any changes to any items; none of the suggested changes were consistent or considered important for accurate measurement and therefore no changes were made to the item wording. All participants considered the 2 global HRQoL items from EORTC QLQ-C30 to be relevant to their experience of mSS, and no changes to these items were suggested.

### Conceptual coverage of selected PRO items and impact domains

A good level of conceptual saturation was achieved; of the 39 symptom concepts reported, 32 (82.1%) were reported spontaneously in the first 2 of 3 saturation groups. Similarly, 8 of the 9 impact domains were reported spontaneously in the first 2 saturation groups.

## Discussion

This study was undertaken to address the gap in qualitative patient research in mSS and to better understand patient experiences of the symptoms and impacts of this disease. Another key objective of this research was to examine the content validity and relevance of a potential new PRO measure (EORTC IL31) designed to be used alongside a well-established cancer PRO measure (EORTC QLQ-C30) in a clinical trial setting. The rare nature of mSS, and the debilitating nature of the condition, means that the evidence in the literature relating to the patient experience of mSS is limited. Indeed, this is the first qualitative research study known to these authors to focus on patients with this specific form of STS.

Previous studies of HRQoL involving patients with SS have examined a broader range of patients with other forms of sarcoma—without presenting data specifically for any SS patients that this included—and have primarily been quantitative studies, often collecting data using existing measures, not exploratory qualitative research [8–12, 14, 18]. For instance, the SABINE study examined symptoms and impact among 116 patients with metastatic STS or bone sarcoma, including 14 patients (12.1%) with SS. In the SABINE study, patients with STS reported frequent fatigue, insomnia, and pain, with the greatest negative impact of the disease being seen in role functioning and social functioning domains, as measured by the EORTC QLQ-C30 functional scale [8]. Gough et al. examined symptom burden in 113 patients with advanced STS (including just 3 patients [2.7%] with SS) using the Memorial Symptom Assessment Scale-Short Form (MSAS-SF) and found the most common and most troubling symptom impacts to be worry, nervousness, sadness, pain, lack of energy, and difficulty sleeping [11].

Studies have also examined the impact of treatment in patients with advanced STS [18]. For example, a literature review of qualitative research in STS noted concepts of elevated anxiety/depression and issues relating to treatment side effects to be present [10]. Poveda et al. measured HRQoL using EORTC QLQ-C30 in 28 patients with advanced STS (including 3 patients [10.7%] with SS) before and after treatment [9]. The PALETTE trial used EORTC QLQ-C30 to assess the impact of treatment on HRQoL in 369 patients with advanced STS (including 44 patients [11.9%] with SS) [12, 19]. The ongoing HOLISTIC study is collating HRQoL data on patients with advanced or metastatic STS undergoing chemotherapy, using a battery of PRO measures including EORTC QLQ-C30; this cohort will also likely include a small proportion of patients with SS [18]. However, as mentioned previously, STS represents a heterogenous group of malignancies, and since only a small proportion of the samples above had a diagnosis of SS and data specifically in this subgroup were not detailed separately, these studies provide limited insight into patients with this particular form of sarcoma.

Another reason for the lack of data on PROs in mSS is the absence of a specific PRO measure for use in these patients. Skalicky et al., noticing a similar gap for sarcoma-specific PRO tools, had examined a symptom inventory specific to 4 subtypes of soft-tissue sarcoma (leiomyosarcoma, undifferentiated sarcoma, liposarcoma, or SS). However, only 2 participants with SS were included among the 27 patients with advanced primary or metastasized STS in the qualitative study assessing the validity of this scale. As such, the relevance of this tool for patients with mSS is not known [13].

The EORTC IL31 is the first mSS-specific PRO instrument to be developed. Our study confirmed the relevance and content validity of the EORTC IL31 when used as an add-on to the EORTC QLQ-C30 for patients with mSS, thereby supporting their combined use in a clinical trial setting to support secondary and exploratory endpoints.

Use of these tools in combination showed that while patients with mSS shared some of the symptoms seen in the wider STS setting, such as fatigue, insomnia, pain, and anxiety, there were additional symptoms and impacts unique to patients with mSS. For example, shortness of breath emerged as a key issue for patients, both in terms of frequency and impact, which may be related to the high incidence of lung metastases in this population. Indeed, this qualitative research suggested that patients with mSS experience numerous symptoms across domains of pain, difficulty breathing, fatigue, and gastrointestinal. These symptoms impact their lives across 9 core domains of functioning/HRQoL, clarifying the specific patient experiences to consider when establishing a PRO strategy in an mSS clinical trial setting. Some of the symptoms reported in this study may be considered a side effect of treatment, including gastrointestinal symptoms, shortness of breath, and fatigue. However, it was not feasible to capture the timing of first symptom appearance during interviews, precluding any definitive link.

A link was identified between the most common symptoms and those considered most important by patients, with the exception of nausea and diarrhea, which were uncommon but highly impactful symptoms. In the results we have distinguished between symptoms and impacts that were spontaneously reported and those only reported when probed (asked about directly). Those spontaneously reported are likely to be the symptoms that have the greatest impact on a patient’s life and thus give insight into their relative importance to this patient population.

Qualitative evidence from these interviews supports the content validity and relevance of the selected PRO items for measuring the most important symptoms and impacts on functioning/HRQoL of mSS in a clinical study setting. The majority of symptoms reported by participants, and in particular those concepts most commonly reported in the sample, are included in EORTC QLQ-C30 and EORTC IL31. Furthermore, most of the items included in the PRO measures were relevant, with no significant gaps identified that warranted revisions.

Although these PRO measures do not measure pain specifically in all locations reported, capturing each of the varied pain locations with individual questions is impractical, so a more general pain item was considered acceptable. Items assessing personal care and cognition were of less relevance for this patient population.

This study has a number of potential limitations. Firstly, the sample size was small, reflecting the rare nature of mSS and the poor health of patients. Although a slightly larger sample would have been preferable and would have given greater confidence that saturation was achieved, it was still judged that saturation had been achieved in the sample of 8 participants. Consistency between the key symptoms reported in the CE part of the interview and the relevance of the items discussed in the CD section also suggested that the sample provided consistent and reliable information. Secondly, the single-site design of the study may have introduced some bias, although sampling quotas implemented should have mitigated against this possibility. Thirdly, the study was limited to US participants from southern California who spoke English. Although the group was diverse in terms of demographic and clinical characteristics, including age, sex, and sites of metastases, there was only one participant aged < 30 years, which may be related to the inclusion/exclusion criteria of this study. Lastly, the length of the interview limited the ability to debrief all items in detail with all participants and required a focus on confirming relevance without in-depth exploration of understanding. However, a longer interview was considered unsuitable due to the nature and severity of this condition, and since the items were taken from the EORTC Item Library, it has already been established that they are well understood.

## Conclusions

Understanding the patient experience of mSS is important; this study provides valuable insight into this previously understudied area. The EORTC IL31 Disease Symptoms PRO instrument is based on preexisting, validated cancer instruments and covers symptoms considered most likely to be relevant to the patient population based on literature review and clinical input.

This study confirmed the content validity of the EORTC IL31 and validated the EORTC QLQ-C30 for assessing patients with mSS. Together, the selected PRO items assess all of the most relevant and important symptoms and impact concepts for mSS, with no notable gaps, supporting their combined use in a clinical trial setting to help assess the impact of treatments on PRO measures of importance to patients.

## Supplementary Information

**Additional file 1.** Literature review details and participant descriptors for symptoms and impacts.

## Data Availability

GSK makes available anonymized individual participant data and associated documents from interventional clinical studies which evaluate medicines, upon approval of proposals submitted to www.clinicalstudydatarequest.com. To access data for other types of GSK sponsored research, for study documents without patient-level data and for clinical studies not listed, please submit an enquiry via the website.

## Abbreviations

mSS: Metastatic synovial sarcoma
PRO: Patient-reported outcome
EORTC IL31: European Organization for Research and Treatment of Cancer Item Library 31
QLQ-C30: EORTC Quality of Life Questionnaire Core 30
CE: Concept elicitation
CD: Cognitive debriefing
SS: Synovial sarcoma
STS: Soft-tissue sarcoma
HRQoL: Health-related quality of life
SF-36: Short-Form-36
EQ-5D: EuroQoL 5-dimension scale
EORTC QLQ-C30: European Organization for Research and Treatment of Cancer Quality of Life Questionnaire Core 30
MDASI: MD Anderson Symptom Inventory
MSAS: Memorial Symptom Assessment Scale
EORTC QLQ-CIPN20: EORTC Quality of Life Questionnaire – Chemotherapy-Induced Peripheral Neuropathy 20-item scale
EORTC QLQ-LC13: EORTC Quality of Life Questionnaire – Lung Cancer module
ADL: Activities of daily living
MSAS-SF: Memorial Symptom Assessment Scale-Short Form
