# Supplementary material for: Qualitative study to characterize patient experience and relevance of patient-reported outcome measures for patients with metastatic synovial sarcoma
Source: J Patient Rep Outcomes. 2022 May 4;6:43. doi: 10.1186/s41687-022-00450-1 (PMC9068846; doi:10.1186/s41687-022-00450-1)
Supplement: Supplementary file 1 — Additional file 1. Literature review details and participant descriptors for symptoms and impacts. [file 41687_2022_450_MOESM1_ESM.docx]

# Additional file 1

## Literature review to explore symptoms and impacts of mSS

A targeted review of existing qualitative research (including a review of MEDLINE, EMBASE, and PSYCHINFO databases) and gray research (including Google Scholar, relevant conference proceedings from the past 2 years, clinicaltrials.gov, and online blogs written by patients with synovial sarcoma [SS]) was conducted to explore symptoms, impacts, and overall experience of patients with SS prior to development and testing of the European Organization for Research and Treatment of Cancer Item Library 31 (EORTC IL31). A total of 12 relevant studies were identified as relevant and reviewed in depth, with relevant content extracted. These revealed that the majority of symptoms reported by patients with metastatic SS could broadly be categorized as pain, breathing/respiratory symptoms, and fatigue/weakness. Weight loss was another symptom reported in the clinical literature, and a range of additional symptoms suggestive of treatment side effects were mentioned in patient blogs. None of the published sources discussed the impact of disease on domains of functioning or health-related quality of life; however, the impact of symptoms on physical function, leisure activities, and emotional health were mentioned in patient blogs. The literature research informed the initial selection of the items for the SS patient-reported outcome (PRO) measures. The search also confirmed the lack of existing SS-specific PRO measure.

## Table S1. Participant Descriptors for Specific Symptom Items

| Descriptor | n | Example Quote |
| --- | --- | --- |
| **Nerve/shooting/burning pain** (n=7 participants) | | |
| Shooting | 5 | *“I do have a shooting pain in my fingers and hands. Well, just in my right hand.”* (03-M-55) |
| Nerve | 4 | *“So that nerve pain would be kind of like the—like a spasm to me and those can be pretty painful.”* (05-M-61) |
| Spasm/cramp | 2 | *“That's kind of like what I was associating like the leg cramp. Those are, those are spasms that I have.”* (05-M-61) |
| Sharp | 2 | *“And I get these sharp pains in my right shoulder in front...* *it's part of the surgery, the nerve system*.*”* (14-M-36) |
| Sciatica | 1 | *“…one of the tumors in the pelvic area, um, was affecting the sciatic nerve and I was having a lot of sciatica pain…”* (12-F-43) |
| Phantom | 1 | *“…I feel that in my right arm where the, uh, sarcoma originated. Um, so I feel the phantom pain…”* (09-M-36) |
| Numb | 1 | *“Um, my right hand, it's, it's numb from my pinkie all the way to my forearm, um, extreme…It affected the nerves or something.”* (14-M-36) |
| **Arm/shoulder pain** (n=5 participants) | | |
| Shooting pain | 2 | “*…it just kind of shoots up to my muscle—to my shoulder…”* (01-F-21) |
| Numb | 2 | *“…it's always numb…”* (03-M-55) |
| Muscle/cramping pain | 1 | “*…kind of as if my muscle were to be cramping and it just kind of shoots up to my muscle—to my shoulder…”* (01-F-21) |
| Phantom pain | 1 | *“I have phantom pain. Uh, 'cause my arm was amputated.”* (09-M-36) |
| Sharp pain | 1 | *“I get these sharp pains in my right shoulder in front.”* (14-M-36) |
| **Chest pain** (n=4 participants) | | |
| Chest pain | 4 | *“…it makes me feel pain a little in my chest…”* (03-M-55) |
| Lung pain | 1 | *“[And would you say the chest pain is similar or, or different from the pain in your lungs and having trouble breathing?] Um, I want to say it's similar.”* (01-F-21) |
| Chest pressure | 1 | *“…it's pressing my, my chest a little bit.”* (03-M-55) |
| **Fatigue** (n=7 participants) | | |
| Fatigue | 3 | *“I have fatigue…”* (03-M-55) |
| Weak | 2 | *“I've been kind of, you know, weak, weaker before…”* (03-M-55) |
| Tired | 2 | *“I get pretty tired…”* (09-M-36) |
| Less energy | 2 | *“Um, like, like little to no energy.”* (05-M-61) |
| Sluggish | 1 | *“Um, I felt sluggish.”* (05-M-61) |
| Worn out | 1 | *“It wears me out. It wears me out.”* (05-M-61) |
| Lethargic | 1 | *“Um, lethargic.”* (13-F-46) |
| **Additional symptoms** (n=8 participants) | | |
| Hair loss* | 2 | *“…some medications have made me lose my hair…”* (12-F-43) |
| Mouth/tongue sores* | 2 | *“…tongue is affected because it has little like small like fissures, cuts.”* (12-F-43) |
| Change in weight | 2 | *“I'm trying to gain some weight actually. I've been—I lost so much weight it was ridiculous.”* (05-M-61) |
| Difficulty swallowing | 1 | *“…when I drink water or something or liquids…I can't swallow quick.”* (03-M-55) |
| Sore throat | 1 | *“…a little bit of a sore throat…”* (12-F-43) |
| Hand and foot syndrome* | 1 | *“…hand and foot syndrome where calluses form on your hands and your feet and, uh, they could be painful.”*  (08-M-56) |
| Infection* | 1 | *“…one port…was disintegrating apparently. And it got infected. So they had to take that port out.”* (05-M-61) |
| High blood pressure* | 1 | *“…I'll have extremely high blood pressure.”* (05-M-61) |
| Blisters* | 1 | *“…my feet, they got—they blistered, both feet blistered…”* (05-M-61) |
| Problems with urination* | 1 | *“…having to wear, um, maxi pads...because, um, you know, the pee goes out if I get a really bad hacking cough.”* (12-F-43) |
| Some participants used more than one descriptor; hence the total exceeds the total number of participants describing the symptom. *Symptoms first experienced after treatment. | | |

## Table S2. Participant Descriptions of Impacts Reported in at Least 50% of Participants, per Domain

| Aspect of domain | Key findings | Associated symptoms | Example quotes |
| --- | --- | --- | --- |
| PHYSICAL FUNCTIONING | | | |
| Difficulty walking  (n=8/8; 100%) | **Description of impact**   - Participants reported that their walking was impacted by their mSS symptoms in terms of: - Generally being more difficult to walk (n=7) - Stopping/avoiding walking (n=5) - Taking more breaks (n=1) | - Bone pain (n=4) - Shortness of breath (n=3) - Hip pain (n=2) - Muscle pain (n=1) - Leg pain (n=1) - Foot pain (n=1) - Back pain (n=1) - Blisters (n=1) - Hand and foot syndrome (n=1) | - *“Bone pain, yeah. Sometimes when I'm walking…Like I'm walking and I want to, I want to break my, my, my feet like oh man that hurts, like ouch.”* (03-M-55) - *“…like if I have to walk to, uh, my mailbox...I just get a lot of shortness of breath or I, I just have to take a, take a break.”* (01-F-21) - *“It was like impossible to walk. You know, painful to, you know, really painful to walk…And, you know, my feet, they got—they blistered, both feet blistered.”* (05-M-61) |
| Needing to rest/sit down  (n=7/8; 87.5%) | **Description of impact**   - Participants described often needing to rest or sit down as a result of their mSS symptoms. Descriptors included: - Needing to sit down (n=6) - Needing to rest (n=5) - To help symptoms reside (n=4) - Needing to go to bed (n=1) | - Fatigue (n=3) - Shortness of breath (n=2) - Bone pain (n=2) - Hip pain (n=1) - Blisters (n=1) | - *“…when I experience like I'm tired, I usually—it's like I sit down and I take like a nap sitting down and then like 30–40 minutes later I feel better.”* (12-F-43) - “…*I sit down or I stretch, you know, my legs. I rub my leg or my whatever is hurting…”* (03-M-55) |
| Difficulty with exercise  (n=5/8; 37.5%) | **Description of impact**   - Stopping/avoiding exercise (n=5)   **Exercise affected**   - Working out (n=2) - Running (n=1) | - Muscle pain (n=1) | - *“I don’t have any exercise, yeah, I mean sometimes it hurts. I guess that's another pain.”* - *“A little pain, you know, sometimes 'cause you don’t use your muscles like you should use them.”* (03-M-55) - *“I want to work out or lift some weights, but I don’t know—yeah, I don’t know if that's the right thing to do.”* (14-M-36) |
| SLEEP | | | |
| Reduced sleep quality  (n=5/7; 71.4%) | **Description of impact**   - Participants reported having a reduced quality of a sleep as a result of their mSS symptoms. - Three participants related their poor sleep quality to treatment (n=3/5). | - Arm/shoulder pain (n=1) - Leg pain (n=1) - Coughing (n=1) - Increased frequency of bowel movements (n=1) - Urgency of bowel movements (n=1) - Diarrhea (n=1) | - *“I can't get a good sleep at night…And, you know, because I'm in pain a little bit here. Then everything—it's like a combination, a combination of pain like in my leg, my arm.”* (03-M-55) - *“So I don’t really sleep well. I kind of—the biggest issue is, um, this cough.”* (12-F-43) - *“…when you're going through treatment…you're not sleeping very well.”* (13-F-46) |
| ACTIVITIES OF DAILY LIVING | | | |
| Difficulty completing household chores  (n=6/7; 85.7%) | **Description of impact**   - Participants avoided doing household chores (n=4). - Participants reported difficulty completing household chores (n=3). - One participant reported needing to take breaks while completing household chores (01-F-21).   **Household chores impacted**   - Cooking (n=3) - Cleaning (eg, washing dishes, sweeping, and mopping) (n=2) - Taking out the trash (n=1) - Grocery shopping (n=1) | - Shortness of breath (n=2) - Fatigue (n=1) - Coughing (n=1) - Neck pain (n=1) - Hand/finger pain (n=1) | - *“I try to be as helpful, you know, in the household as I can. Um, but there's times where I just, um, I don’t have any energy.”* (05-M-61) - *“…like sweeping and mopping, um, I do have to stop throughout it, um, to take a breath…”* (01-F-21) - *“…I don’t feel that good to cook because I'm coughing…”* (12-F-43) - *“…you know, putting groceries in a car because I was already out of breath…”* (13-F-46) |
| Emotional impacts | | | |
| Worry/anxiety  (n=5/7; 71.4%) | **Description of impact**   - Participants reported feeling worried or anxious as a result of their mSS (n=5). - Worry (n=3) - Anxiety (n=2) - Panic (n=1) - Two participants reported being worried about the impact of their mSS on their family and friends (n=2). - One participant reported being worried about dying (12-F-43). - One participant reported being worried about their cancer coming back (13-F-46). | - Participants did not discuss symptoms associated with this impact. | - *“...my anxiety has definitely been shooting through the roof, as well as having many panic attacks...”* (01-F-21) - *“...I worry about all my friends and family who worry about me.”* (05-M-61) - *“...so at the beginning, you know, I was really panicked, had a lot of anxiety because, you know, when you’re diagnosed with cancer you just think you’re going to die. Like you just have days and hours.”* (12-F-43) - *“...you always worry if it’s ever going to come back.”* (13- F-46) |
| Social impacts | | | |
| Impacts on relationships with family/friends/partner  (n=6/6; 100.0%) | **Description of impact**   - Participants reported how their social relationships had been negatively impacted as a result of their mSS (n=6):   - Stopping/avoiding spending time with family/friends/partner (n=4)   - Spending less time with family/friends/partner (n=4)   - Difficulty spending time with family/friends/partner due to emotional impacts (n=2) - Five participants related their negative impacts to their social relationships to treatment (n=5). | - Fatigue (n=2) - Hair loss (n=1) - Weakness (n=1) - Pain (not specified) (n=1) - Coughing (n=1) | - *“...I mean the only time that I really didn’t, you know, go to family activities was when I had lost my hair, you know, or if I feel weak, like tired because I've been on so many different drugs, um, for different clinical trials...”* (12-F-43) - *“I have close friends that I spend time with, I would have to say that I've spent less and less time with them, um, over the past, uh, let's say six months to a year, and a lot of that simply is because of this fatigue from a long day going down there.”* (05-M-61) |
| Diet/eating | | | |
| Changes to diet  (n=5/6; 83.3%) | **Description of impact**   - Participants reported making changes to their diet due to their mSS (n=5). - One participant reported making changes to their diet due to treatment (n=1/5). | - Mouth/tongue sores (n=2) - Nerve/shooting/burning pain (n=1) - Chest pain (n=1) | - *“...on this new medication my, um, tongue is affected because it has little like small like fissures, cuts. So there's—like I can't drink certain things or eat certain foods because it's not—even like the toothpaste, it burns because of the peppermint.”* (12-F-43) - *“...if I had a loss of appetite of the food that I usually eat on a daily basis, well I would find some—I would find different foods that I would—could be able to eat. Like for example, if I couldn’t eat...my mother would make something, uh, healthy or something in the blender for me to at least drink it so I could have some nutrients in my body.”* (13-F-46) - *“...hot like coffee or something cold, once it goes down my throat, I can feel it's kind of hitting me. It's, it's like a trigger...I'm having those, those, uh, you know, those attacks if you may...The shooting pain in my chest...****So you, you just avoid drinking that as much as possible then?*** *When I have it, yes...”* (14-M-36) |
| Loss of appetite  (n=5/6; 83.3%) | **Description of impact**   - Participants reported experiencing a loss of appetite due to their mSS (n=5). - One participant related their loss of appetite to treatment (n=1/5). | - Pain (not specified) (n=1) - Fatigue (n=1) - Mouth/tongue sores (n=1) | - *“...due to the pain that I have, I won't, I won't eat as much...”* (01-F- 21) - *“...while you're doing treatment...uh, you lose your taste buds and you don’t really feel like eating...because you can't swallow anything because you might have mouth sores.”* (13-F-46) - “*I don’t have a lot of, uh, say appetite...”* (08-M-56) |

mSS, metastatic synovial sarcoma.
